# Genome-wide DNA methylation meta-analysis in the brains of suicide completers

**DOI:** 10.1038/s41398-020-0752-7

**Published:** 2020-02-19

**Authors:** Stefania Policicchio, Sam Washer, Joana Viana, Artemis Iatrou, Joe Burrage, Eilis Hannon, Gustavo Turecki, Zachary Kaminsky, Jonathan Mill, Emma L. Dempster, Therese M. Murphy

**Affiliations:** 1 University of Exeter Medical School, University of Exeter, Exeter, UK; 2 Rush Alzheimer’s Neurodisease Center, Rush University Medical Center, 600 South Paulina Street, Chicago, IL 60612 USA; 3 Douglas Institute, Department of Psychiatry, McGill University, Verdun, QC H4H 1R3 Canada; 4 Department of Psychiatry, School of Medicine, Johns Hopkins University, Baltimore, MD USA; 5 Department of Mental Health, Johns Hopkins Bloomberg School of Public Health, Baltimore, MD USA; 6 School of Biological and Health Sciences, Technological University Dublin, City Campus, Dublin, 2 Ireland

**Keywords:** Epigenetics and behaviour, Molecular neuroscience

## Abstract

Suicide is the second leading cause of death globally among young people representing a significant global health burden. Although the molecular correlates of suicide remains poorly understood, it has been hypothesised that epigenomic processes may play a role. The objective of this study was to identify suicide-associated DNA methylation changes in the human brain by utilising previously published and unpublished methylomic datasets. We analysed prefrontal cortex (PFC, *n* = 211) and cerebellum (CER, *n* = 114) DNA methylation profiles from suicide completers and non-psychiatric, sudden-death controls, meta-analysing data from independent cohorts for each brain region separately. We report evidence for altered DNA methylation at several genetic loci in suicide cases compared to controls in both brain regions with suicide-associated differentially methylated positions enriched among functional pathways relevant to psychiatric phenotypes and suicidality, including nervous system development (PFC) and regulation of long-term synaptic depression (CER). In addition, we examined the functional consequences of variable DNA methylation within a PFC suicide-associated differentially methylated region (*PSORS1C3 DMR*) using a dual luciferase assay and examined expression of nearby genes. DNA methylation within this region was associated with decreased expression of firefly luciferase but was not associated with expression of nearby genes, *PSORS1C3* and *POU5F1*. Our data suggest that suicide is associated with DNA methylation, offering novel insights into the molecular pathology associated with suicidality.

## Introduction

Suicide represents a global public health problem, with approximately 800,000 people dying worldwide from suicide annually and suicide attempts up to 20 times more frequent than completed suicide^1^. Moreover, suicide is the second leading cause of death among young people worldwide and ranks among the 20th leading causes of death across all ages^2^. The risk for suicidal acts is multifactorial, and consists of a range of biological, psychiatric, psychosocial, and cultural risk factors^3^. Despite its economic and social burden, the underlying biological aetiology of suicidal behaviour (SB) remains poorly understood.

To date, large-scale genome-wide association studies (GWAS)^4–7^ have failed to identify robust associations suggesting that the risk of SB is highly polygenic in nature and that individual gene variants are likely to account only for a small proportion of the total phenotypic variability^8^. Other factors, such as the environment, behavioural traits, psychiatric diagnosis, lifestyle, and coping mechanisms, are essential regulators of suicide risk and likely to account for more sizeable effects^9^. Recently, increased understanding of epigenetic processes that occur in the brain has opened promising avenues in suicide research. The epigenome is potentially malleable—changing with age^10^, in response to specific environmental^11^ and psychosocial factors^12^—providing a mechanism for the interaction between genotype and the environment^13^. Epigenetic processes, including DNA methylation, have recently been implicated in the aetiology of numerous mental health disorders^14–21^ and SB^22,23^.

In the last decade, research aiming to understand the contribution of epigenetic mechanisms to SB has implicated the role for key biological pathways, including hypothalamic pituitary adrenal axis, stress response, polyamine system, neurotrophic signalling, and lipid metabolism^8^. However, studies examining DNA methylation differences associated with SB have primarily focussed on candidate genes^24–27^ and few have examined genome-wide DNA methylation changes in the brains of suicide completers^22,28^. The availability of brain samples is a major challenge for psychiatric research and many previous studies examining DNA methylation variation in suicide are performed on a limited number of post-mortem brain samples^22,29,30^. Such small studies have reduced statistical power to detect small changes in DNA methylation. The objective of this study was to identify suicide-associated DNA methylation changes in the human brain by utilising previously published and unpublished methylomic datasets.

Genome-wide DNA methylation profiles were available from post-mortem brain samples of suicide completers and non-psychiatric, sudden-death controls for a total of seven cohorts. Methylomic data available for two different brain regions —prefrontal cortex (PFC) and cerebellum (CER) (PFC: 4 cohorts, *n* = 211; CER: 3 cohorts, *n* = 114)—were meta-analysed across the suicide cohorts for each brain region separately. We report evidence for altered DNA methylation in suicide cases compared to non-psychiatric controls in both the PFC and CER and examined the functional implications of a top-ranked PFC suicide-associated differentially methylated region (DMR) on gene expression levels in that region. Finally, gene ontology enrichment analysis was performed in each brain region separately to identify pathways of genes associated with suicide completion.

## Materials and methods

### Sample collection/data recruitment

For the PFC meta-analysis, we included four independent previously published studies^22,31–33^ aimed at profiling DNA methylation in human PFC in individuals with a diagnosed axis-I psychiatric disorder and healthy non-psychiatric controls. Only data from individuals who died by suicide and non-psychiatric controls were included for the initial meta-analysis. In two of the four studies selected^31,32^, DNA methylation was profiled from fluorescence-activated nuclei sorted neurones, with the remaining two studies performed in bulk tissue^22,33^. Raw DNA methylation data for all four studies are deposited in the Gene Expression Omnibus (GEO) database (accession number: GSE89707, GSE88890, GSE98203, GSE41826) and full details of the sample cohort can be obtained from the original studies^22,31–33^. For the CER meta-analysis, three DNA methylation datasets were included two of which are currently unpublished (GSE137222 and GSE137223). Raw DNA methylation data for the CER published EWAS study^33^ is deposited in GEO database (accession number: GSE89702). The unpublished studies were approved by the University of Exeter Medical School Research Ethics Board (REB). In all three CER cohort’s DNA methylation profiles were derived from bulk brain tissue and cases were individuals who died by suicide (hanging, jumping from height, intentional poisoning, self-harm/bleeding). Cause and manner of death as well as joint presence of psychiatric diagnosis were determined by a forensic pathologist after evaluating autopsy results, circumstances of death, data from extensive toxicological testing, police reports, family interviews, and medical records. Controls were individuals who died suddenly (e.g. cardiac failure, viral infection, or accidents) and did not have evidence of axis-I disorders. See Supplementary Table S1 for a complete description of sample selection, numbers, and demographic characteristics of each cohort.

### DNA methylation analysis

DNA methylation was measured using the Illumina HumanMethylation450K BeadChip (‘Illumina 450K array’) or Infinium MethylationEPIC BeadChip (‘Illumina EPIC array’, one CER cohort) platform (Illumina Inc., San Diego, CA, USA). To ensure consistency of the methodological approach, raw DNA methylation data (idat files) were recovered and each cohort was independently reanalysed, applying the same quality control (QC) and pre-processing pipelines. Briefly, QC checks, quantile normalisation, and separate background adjustment of methylated and unmethylated intensities of type I and II probes were employed using the wateRmelon package in R^34^. Probes on the X- and Y-chromosomes were used to confirm sample sex. Only samples which passed stringent QC measures (>1% of sites with a detection *P* value (*P*) >0.01) were included. Probes with a detection *P* > 0.01 in at least 1% of samples and/or a beadcount <3 in 5% of samples, non-specific probes, potentially cross-reactive probes, or probes near SNPs^35,36^ were removed across all samples. Only probes common to both the 450k array and EPIC array were included in downstream analyses for the CER. For the annotation of probes, the University of California, Santa Cruz (UCSC) RefGene name from Illumina’s annotation file and enhanced annotation to the UCSC Known Gene were used. All annotations used the human February 2009 (GRCh37/hg19) assembly.

### Estimating differential neuronal proportions

The R package (available at www.cran.r-project.org), Cell EpigenoType Specific (CETS) mapper, designed for the quantification and normalisation of differing neuronal proportions in genome-wide DNA methylation datasets was used as previously described^32^ to estimate brain cellular heterogeneity in each of the four PFC cohorts. Similar estimates could not be obtained for the CER cohorts as the algorithm for the correction of brain cellular heterogeneity bias was developed using post-mortem frontal cortex data and NeuN is not expressed in CER purkinje neurons^32^.

### Data analysis

Statistical analyses were performed using R statistical package (version 3.4.3). The *β-*value is a ratio between methylated probe intensity and total probe intensities (sum of methylated and unmethylated probe intensities) and ranges from 0 to 1. Linear regression was used to examine differences in DNA methylation scores (reported as change in *β-*value (∆*β*)) between suicide cases and controls at each CpG site, controlling for potential confounders. Covariates included in all models were age, sex, and chip. We also included ethnicity or brain bank as covariates in the model for those cohorts where that information was available and represented a potential source of variation. In the PFC cohorts only, we also adjusted for estimated neuronal proportions. For one study^22^, DNA methylation differences were investigated across individual-matched cortical regions (Brodmann area 11 (BA11), Brodmann area 25 (BA25)) by fitting a linear mixed-effect model (LMM) using the lme4 R package (available at https://cran.r-project.org^37^). Whereby, brain region and sample ID were included in the model as random effects (‘within participants’ factors) while diagnosis, age, sex, PH, and cellular composition were included in the model as fixed effects.

### Meta-analyses

#### Suicide completers versus non-psychiatric controls

The results obtained from the linear regression were then meta-analysed for each brain region independently. A fixed-effect model, using the ‘metagen’ function in the R package ‘meta’, was applied by providing the regression coefficients and standard errors from each individual cohort to calculate weighted pooled estimates and to test for significance. Experiment-wide significance (*P* < 1E-07) (threshold estimated from permutation analysis in a larger dataset (*N* = 675 individuals) generated previously by our group^38^) was chosen as a multiple testing threshold to determine statistically significant DNA methylation changes.

#### Suicide completers versus non-suicide psychiatric controls

In order to assess whether the observed suicide-associated DNA methylation changes identified in our original meta-analysis were driven by the psychiatric disorder comorbidity rather than being suicide-specific changes, a second exploratory analysis was performed in additional samples obtained from the CER datasets only, which had additional non-suicide psychiatric samples with DNA methylation data available (not included in the primary analysis report here). In the second meta-analysis, each CER cohort consisted of suicide cases (that were included in the original meta-analysis) and psychiatric controls, where individuals had a diagnosed axis-I disorders (major depressive disorders (MDD), schizophrenia (SZ), bipolar disorder (BD)) but had no documented evidence of SB. In total, the secondary meta-analysis included 130 samples (case group, *N* = 50; psychiatric control group, *N* = 80). Results obtained from the linear regression were then meta-analysed using a fixed-effect model as described previously.

### Region-based analysis

The results obtained from both the PFC and CER meta-analyses were used to perform a regional-based analysis using the Python module Comb-p^39^ to identify suicide-associated DMRs. The Comb-p software groups spatially correlated DMPs (seed *P* < 1E-03, minimum of three probes) at a maximum distance of 500 bp in each brain region. DMR *P* were corrected for multiple testing using Šidák correction^40^.

### Gene ontology term enrichment analysis

A previously described logistic regression approach^41^ was used to test if genes (Illumina UCSC gene annotation) annotated to probes in our PFC and CER meta-analyses (DMPs with *P* ≤ 1E-04) predicted pathway membership, while controlling for the number of probes annotated to each gene. Briefly, pathways were downloaded from the Gene Ontology (GO) website (http://geneontology.org/) and all genes annotated to parent terms were also included. Genes containing at least one Illumina probe and annotated to at least one GO pathway were considered. Pathways were filtered to those containing between 10 and 2000 genes and a list of significant (after correction for multiple testing—Bonferroni correction) pathways were identified as previously described^41^.

### Functional follow-up of significant DNA methylation findings

Tissue (*N* = 71) from two regions of the cortex, BA11 (*N* = 38) and BA25 (*N* = 33), collected from 20 MDD suicide cases and 20 non-psychiatric sudden-death controls was obtained from the Douglas Bell Canada Brain Bank (DBCBB) (http://douglasbrainbank.ca/), further details are available in ref. ^22^. Previously, our group performed DNA methylation profiling in these samples^22^ and the results of that study were included in this meta-analysis study. To examine whether our identified suicide-associated DMR (*PSORS1C3 DMR;* Chr6:31,148,370-31,148,553 (Hg19), 2694 bp downstream the TSS of *PSORS1C3* gene) is associated with the expression of nearby genes, we measured expression levels of two nearby genes in these brain tissue samples. We tested for an association with gene expression firstly at the closest transcription start site (TSS) gene—the lncRNA gene*, PSORS1C3*—and then at the second closest gene*, POU5F1* (see Supplementary Fig. S1 for details).

### Gene expression analysis

Thirty milligrams of frozen PFC tissue from each brain sample was homogenised with Qiazol Lysis Reagent (Qiagen, Valencia, CA, USA), as per the manufacturer’s instructions, before running it through a QIAshredder (Qiagen, Valencia, CA, USA). Total RNA was extracted using the Qiagen miRNeasy Mini column-purification system and treated with DNase I as outlined by the manufacturer. The Agilent 2100 Bioanalyzer was used to check the quality and concentration of the extracted RNA samples. One microgram of total RNA was reverse transcribed into complementary DNA (cDNA) (20 μL reactions) according to the manufacturer’s instructions using the Invitrogen VILO cDNA synthesis kit (Life Technologies Ltd, Paisley, UK). Three housekeeping genes Ubiquitin Conjugating Enzyme E2 D2 (*UBE2D2*), Cytochrome C1 (*CYC1*), and Ribosomal Protein L13 (*RPL13)* identified previously^42^ as being among the most stably expressed in the brain were selected to normalise the target gene expression. Next, quantitative RT-PCR was performed in triplicate for each assay using the StepOnePlus Real-Time PCR machine (Applied Biosystems, Foster City, Calif) and pre-optimised Taqman gene expression assays (Applied Biosystems, Foster City, Calif). A full list of the qPCR assays used is given in Supplementary Table S2. PCR cycling conditions were as follows: 50 °C for 2 min, 95 °C for 20 s, and 40 cycles of 95 °C for 10 s, and 60 °C for 20 s. We undertook stringent QC of raw qPCR data, repeating samples where there was high variability between triplicates (Ct > 0.5). The abundance of each test gene was determined by the comparative Ct method^43^, expressed relative to the geometric mean of the three housekeeping genes. Data were log2-transformed to ensure normal distribution and presented as a fold-difference in expression of suicide cases relative to controls using the 2−ΔΔCT method. To assess whether *POU5F1* expression levels were associated with a history of suicide, we used a LMM using the lme4 R package (available at https://cran.r-project.org)^37^ where ΔCt values of the target gene (*POU5F1*) was the response variable. Brain region and sample ID were included in the model as random effects (‘within participants’ factors) while diagnosis, age, sex, and neuronal proportion were included in the model as fixed effects. Finally, since 450K array data were available from the same individuals, we examined the correlation between gene expression levels and mean DNA methylation levels at the DMR.

### Reporter constructs

The *PSORS1C3 DMR* sequence was inserted into the pCpGL-basic vector (see ref. ^44^ for details), which is devoid of CpG sites and was generously provided by the Rehli laboratory^44^. Briefly, the cleaned *PSORS1C3* PCR amplicon was inserted into a digested pCpGL-basic plasmid using T4 ligase and buffer (Invitrogen, California, USA). Ligated plasmids were transformed into One Shot PIR1 *E. coli* (ThermoFisher Scientific, Massachusetts, USA) to allow for monoclonal amplification of the recombinant plasmids. For the transformation, 50 µl of One Shot PIR1 *E. coli* was used, including a negative ligation control and a positive transformation control (pUC19) using standard procedures.

Clones were subsequently checked by clonal PCR, restriction digest using *Bgl*II and *Nco*I and Sanger sequencing (see Supplementary Fig. S2) to confirm the DMR had been inserted in the correct orientation. The pCpGL construct was methylated in vitro using M.sssI methyltransferase (New England Biolabs, Massachusetts, USA) in the presence of *S*-adenosylmethionine (SAM) following the manufacture’s protocol. An empty pCpGL-basic vector was also methylated to act as a control. To confirm successful methylation, the plasmids underwent digestion with the methylation sensitive enzyme *Hpa*II (see Supplementary Fig. S3).

Cell culture and transfections in HEK293 cells were cultured in Dulbecco’s modified Eagle’s medium (þ4.5 g l_1 d-glucose, l-glutamate, pyruvate) (Gibco) with 10% foetal bovine serum (Gibco) at 37 °C and 5% CO_2_. Briefly, 2 × 10^5^ cells were seeded in six-well plates. The following day media was removed, the cells washed with PBS, and 1.5 ml of fresh growth media was added. Five hundred nanograms of PSORS1C3 or pCpGL plasmid and 100 ng of pGL4.74[hRluc/TK] reporter control vector (Promega, Wisconsin, USA) were diluted in 500 µl of Opti-MEM reduced serum media (Gibco, Massachusetts, USA) in an Eppendorf and left to equilibrate for 5 min at room temperature. In all, 4.5 µl of Lipofectamine LTX Reagent (ThermoFisher Scientific, Massachusetts, USA) was then added to each Eppendorf and incubated for 30 min at room temperature. Following incubation, 500 µl of LTX plasmid mix was added to the HEK293 cells in six-well plates. Cells were then incubated at 37 °C, 5% CO_2_, for 24 h to allow expression of firefly and *Renilla* luciferases.

### Dual-luciferase assay

Twenty-four hours following transfection a dual-luciferase reporter assay (Promega, Wisconsin, USA) was carried out to measure the expression of firefly luciferase and *Renilla* luciferase in the transfected cells as per the manufacturers’ instructions. Each experiment contained three technical repeats and the experiment was repeated three times. The injections and light absorbance were carried out automatically using the pherastar plate reader. The average firefly luciferase activity was calculated by averaging absorbance readings between 2 and 10 s. The average *Renilla* luciferase activity was calculated by averaging absorbance readings between 14 and 22 s. Data analysis was carried out as described in ref. ^45^. All data are presented as a normalised firefly luciferase activity relative to *Renilla* luciferase. Fold change expression was calculated by dividing the unmethylated normalised firefly luciferase activity by the respective methylated normalised luciferase activity. A Student’s *T*-test was used to compare the methylated versus unmethylated vectors.

## Results

### Suicide-associated DMPs in human cortex and cerebellum

**Fig. 1:**
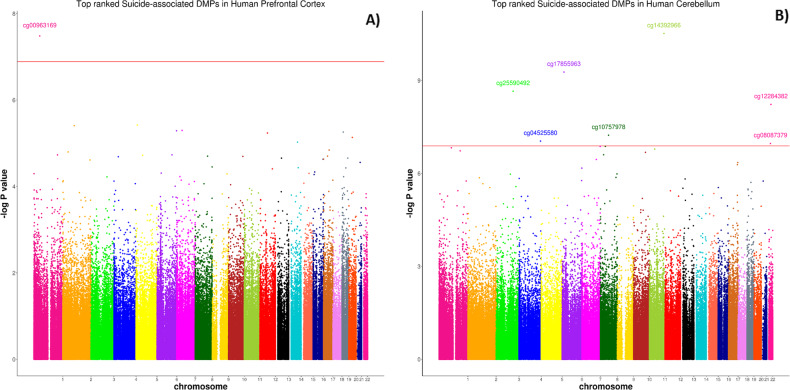
Suicide-associated DMPs in human cortex and cerebellum. Manhattan plot showing site-specific genome-wide pattern of DNA methylation in **a** the human prefrontal cortex (PFC) and **b** the human xerebellum (CER). One CpG site was identified as differentially methylated between suicide cases and healthy controls at experiment-wide significant (*P* = 1E-07) in the PFC. Six CpG sites were identified as differentially methylated between suicide cases and healthy controls at experiment-wide significant (*P* = 1E-07) in the CER.

An overview of the methodological approach used in this study is given in Supplementary Fig. S4. We identified one DMP (cg00963169) in the PFC, which reached experiment-wide significance (*P* = 3.30E-08 (Fig. 1a)). The effect size at this CpG site, located downstream of exon 1 of the neuron-specific protein coding gene, ELAV-like RNA binding protein 4 (*ELAVL4)*, was largely consistent across all cohorts included (see Supplementary Fig. S5), showing hypomethylation in suicide cases relative to controls. Interestingly, the 20 most significant (*P* < 5E-05) suicide-associated differentially methylated loci identified in the PFC, listed in Supplementary Table S3, include probes in the vicinity of several loci previously implicated in psychiatric phenotypes. In the CER, six probes (cg14392966, cg17855963, cg25590492, cg12284382, cg10757978, cg04525580) passed the experiment-wide significance threshold (*P* < 1E-07) (Fig. 1b). Of interest the top-ranked DMP, cg14392966 (*P* = 3.06E-11), which is located within the coding region (exon 1) of the *PUS3* gene on Chr11, has been previously associated with severe neurodevelopmental disorders^46^. Supplementary Figure S6 shows that this DMP is hypomethylated in two of the three cohorts in suicide cases compared to healthy controls. A list of the top 20 DMPs in the CER is provided in Supplementary Table S4*.*

### Region-based analysis of altered DNA methylation in suicide completers

**Fig. 2:**
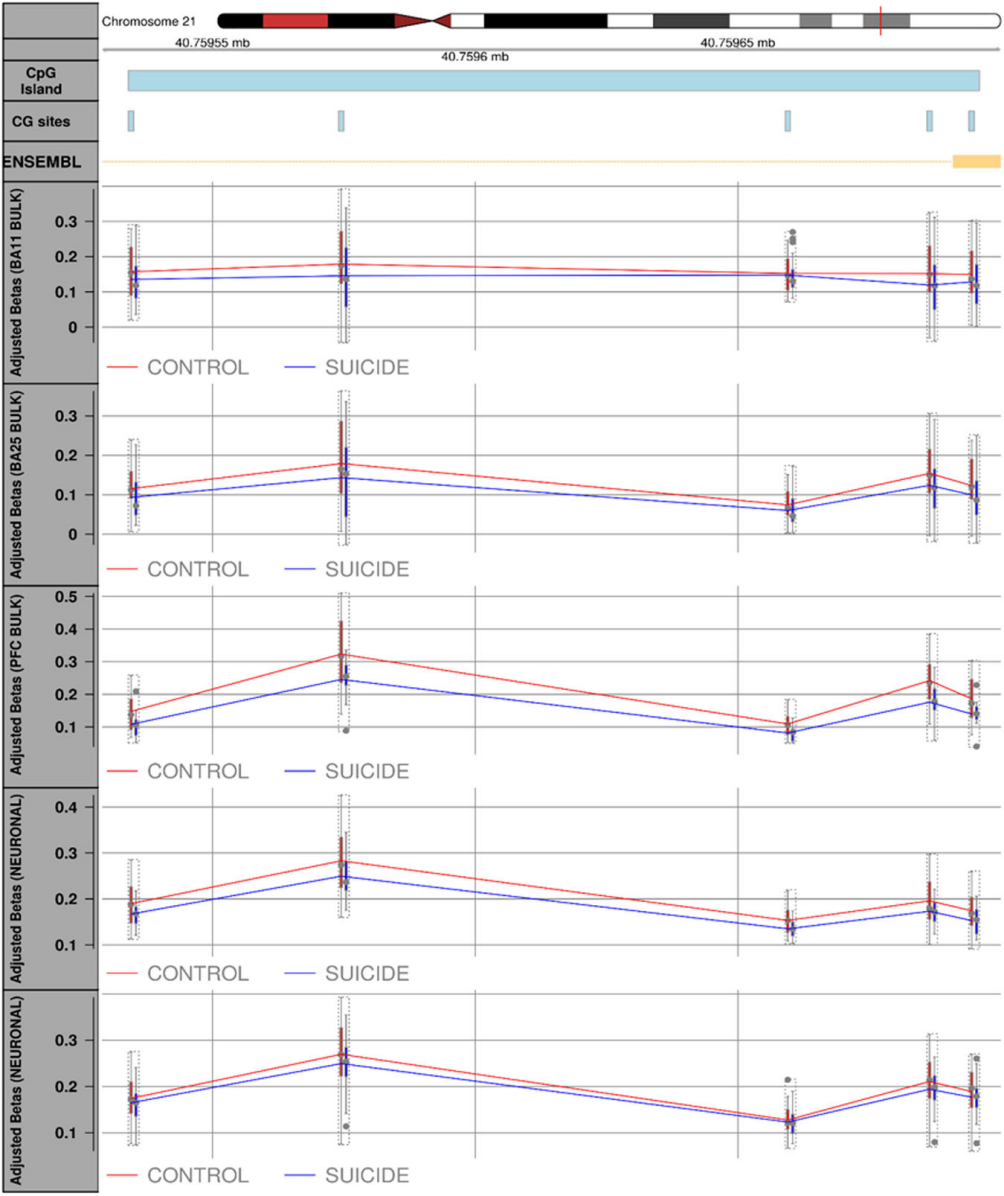
Suicide-associated differentially methylated region (DMR) in human Prefrontal cortex (PFC). Plot showing the top-ranked DMR in the PFC. This DMR, spanning 5 CpG sites and located in the promoter region of the *WRB* gene (Sidak-corrected *P* = 5.11E-06), was found consistently hypomethylated in suicide cases compared to healthy controls, across all 4 suicide brain cohorts. The solid line is for illustration purposes and not indicative that the CpG sites between sites are also methylated.

We used the python module, Comb-p^39^, to identify DMRs in suicide cases compared to controls in each brain region. The regional analysis identified three and eight significant (Sidak-corrected *P* < 0.05) DMRs in the PFC and CER, respectively (see Table 1 for details). In the PFC, the top-ranked DMR was found within the *WRB* gene (Sidak-corrected *P* = 5.11E-06) and was consistently hypomethylated across all five CpG sites in suicide cases relative to controls, in all four PFC methylomic studies (Fig. 2). Of interest, the second top-ranked suicide-associated DMR in the PFC (Sidak-corrected *P* = 3.81E-05) was located downstream of the promoter region of the *PSORS1C3* non-coding gene, a DMR previously reported by our group as associated with MDD suicide completers^22^.

**Table 1:** Comb-p differentially methylated region (DMR) analysis.

Brain region	Hg19	Annotated gene (UCSC)	No. of probes	Slk *P* value	Sidak *P* value
PFC	chr21:40759534-40759695	*WRB*	5	2.02E-09	5.11E-06
	chr6:31148370-31148553	*PSORS1C3*	10	1.71E-08	3.81E-05
	chr22:38071168-38071189	*LGALS1*	3	3.37E-08	0.0006529
CER	chr22:17956453-17956561	*CECR2*	4	1.55E-10	5.68E-07
	chrX:79590789-79590956	*CHMP1B2P*	4	3.74E-09	8.84E-06
	chr13:99100506-99100587	*FARP1*	3	2.68E-09	1.31E-05
	chr6:31838402-31838529	*SLC44A4*	5	2.68E-08	8.32E-05
	chr3:149374761-149374915	*WWTR1*	3	7.56E-08	0.0001938
	chr12:116756805-116756949	*MED13L*	3	8.64E-08	0.0002369
	chr1:1846046-1846155	*CALML6*	3	9.40E-08	0.0003406
	chr11:2397486-2397686	*CD81-AS1*	4	2.10E-06	0.004138

*CER* cerebellum, *PFC* prefrontal cortex, *Hg19* human genome version 19, *UCSC* University of California, Santa Cruz Human Genome Browser. Stouffer-Liptak-Kechris correction (slk); one-step Siidak (1967) multiple testing correction.

**Fig. 3:**
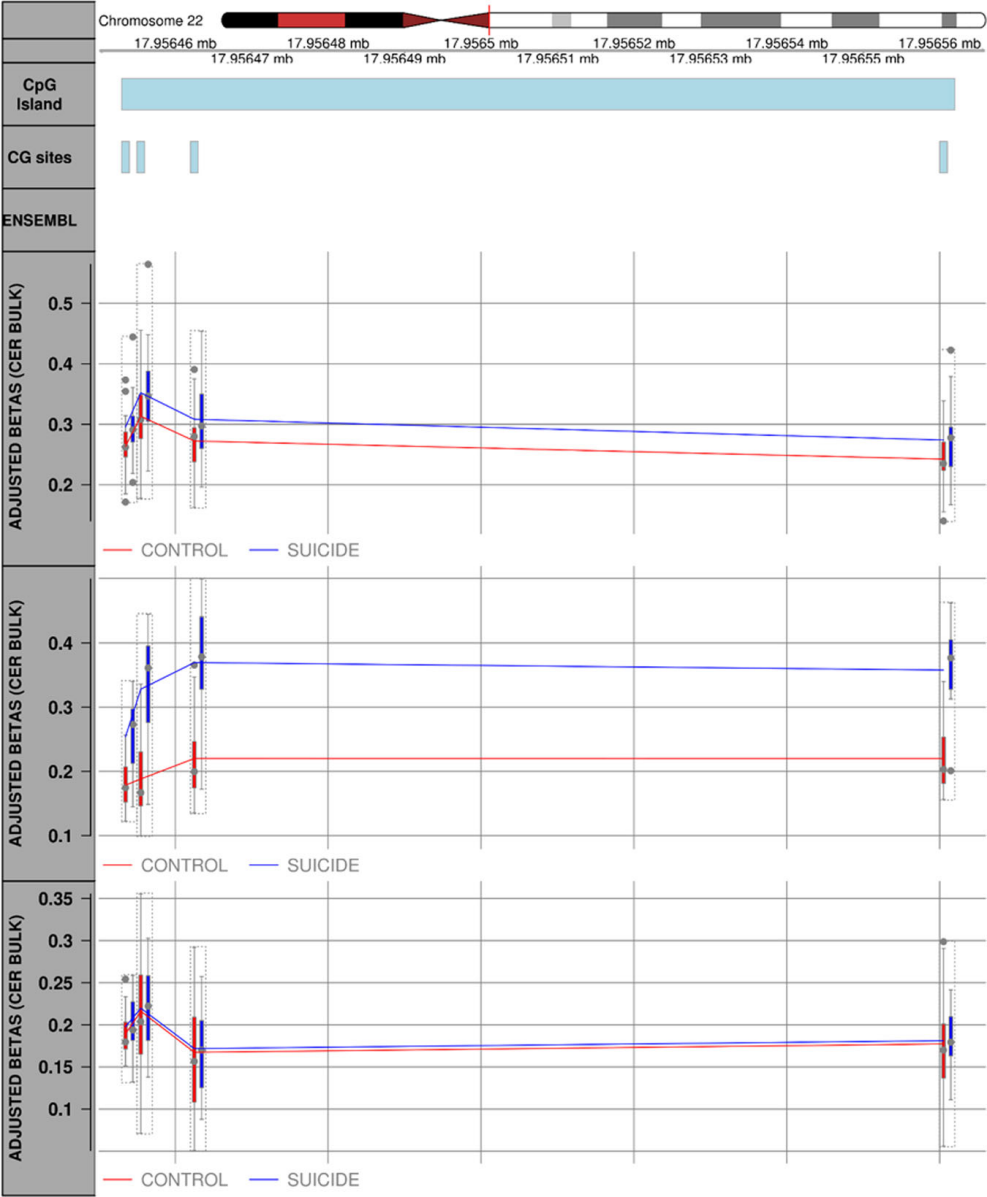
Suicide-associated differentially methylated region (DMR) in human Cerebellum (CER). Plot showing the top-ranked DMR in the CER. This DMR, spanning 4 CpG sites and located within the coding region of the *CECR2* gene (Sidak-corrected *P* = 5.68E-07), was found consistently hypermethylated in suicide cases compared to healthy controls, across 3 suicide brain cohorts. The solid line is for illustration purposes and not indicative that the CpG sites between sites are also methylated.

In the CER, the top-ranked suicide-associated DMR was identified on chromosome 22, distributed along the intronic region of the *CERC2* gene and spanning 4 CpG sites. The *CERC2*-associated DMR (Fig. 3) showed significant hypermethylation (Sidak-corrected P = 5.68E-07) across all 4 CpG sites within the region in suicide cases compared with controls. The direction of this change was found to be consistent across all 3 CER methylomic studies.

### Pathway analysis

The biological relevance of our findings was investigated through gene ontology analysis on genes annotated to suicide-associated DMPs (*P* ≤ 1E-04). Results revealed an enrichment of DNA methylation alterations in genes involved in cognitive processes such as long-term synaptic depression and brain development (See Supplementary Table S5 and S6).

### Suicide-associated DMPs identified in the CER are largely independent of comorbid psychiatric disorders

In order to disentangle the relative contribution of psychopathologies from DNA methylation changes specific to suicide, we performed an additional meta-analysis in the CER whereby all the non-psychiatric controls initially included in our meta-analysis were excluded and individuals with documented Axis-I psychiatric disorders (MDD, SZ, BD) and who died by suicide were compared to psychiatric cases without a documented history of SB/suicide fatalities (3 cohorts, *N* = 130, cases *N* = 50, controls *N* = 80). Comparison of the results (effect sizes at the top 500 (*P* < 0.05) DMPs) from our original CER meta-analysis and the secondary analysis (Supplementary Fig. S7) revealed a strong positive correlation (*P* = 2.2E-16; *R* = 0.89). Moreover, 2 of the 6 CER-associated DMPs (*P* < 1E-07) were nominally significantly differentially methylated in our suicide cases versus psychiatric controls analysis (cg10757978, *P* = 1.19E-04; cg04525580, *P* = 0.017) (see Supplementary Table S7 for details) and a similar direction of effect was observed for both analyses for the remaining 4 CER-associated DMPs. We were unable to perform the same analysis in the PFC due to lack of samples from individuals with an axis-I diagnosis who did not die by suicide.

### Functional validation and gene expression analysis of the PSORS1C3 DMR

Given our replication of a *PSORS1C3 DMR* in suicide^22^ we aimed to functionally evaluate its effect on the expression of nearby genes. First, we examined the effect of DNA methylation at the suicide-associated DMR on nearby gene expression using a CpG-Free Luciferase Reporter (pCpGL vector) gene assay^47^. Next, we examined gene expression levels of nearby annotated genes (*PSORS1C3* and *POU5F1)* in a subset of samples for which brain tissue was available (two brain regions (BA11 and BA25, *N* = 70; suicide cases (*N* = 36), non-psychiatric controls (*N* = 34)) and examined the correlation between expression levels of our selected target genes and mean DNA methylation at the suicide-associated DMR.

**Fig. 4:**
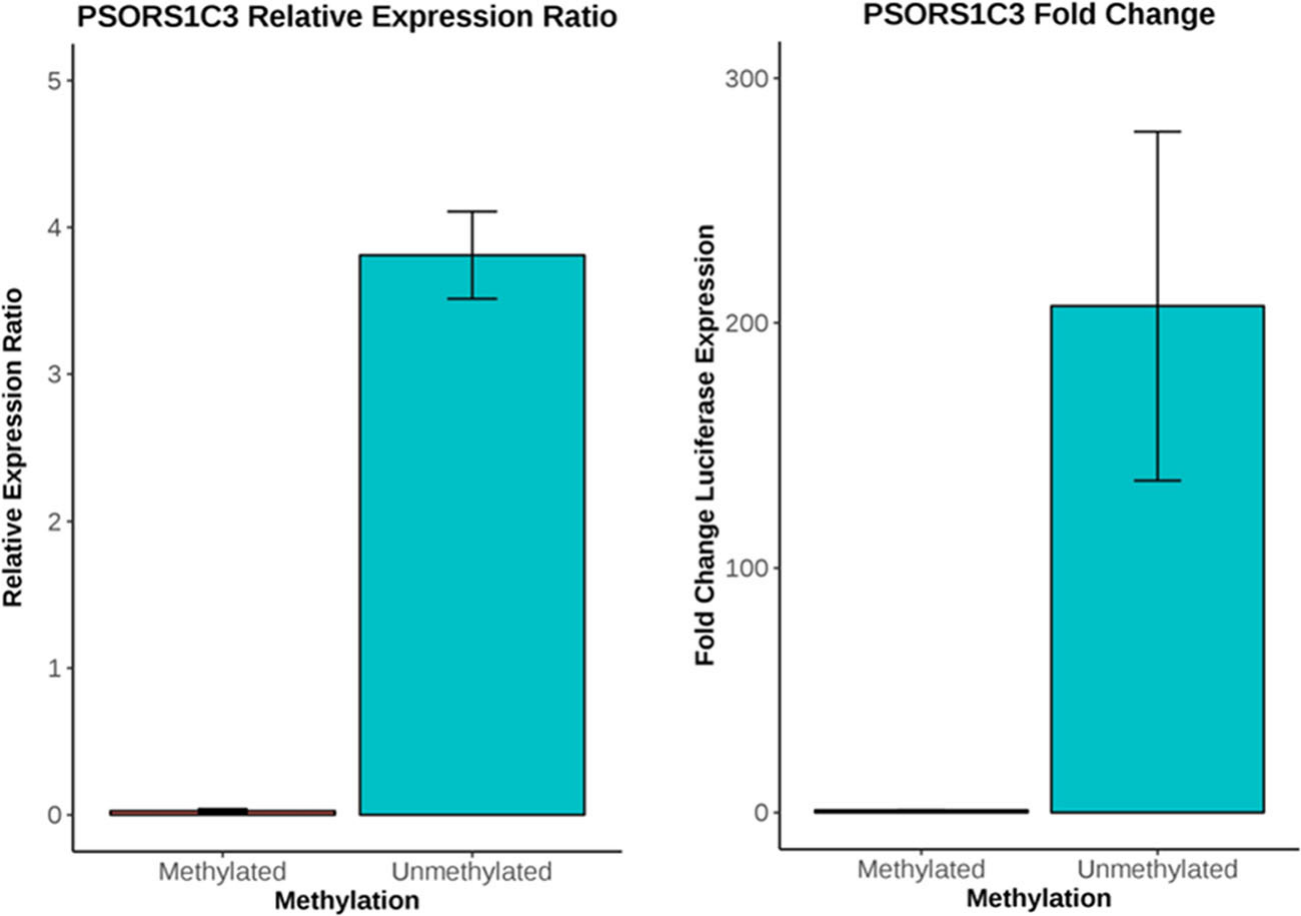
Methylation of the *PSORS1C3* cloned pCpGL vector significantly reduces luciferase activity. There is a significant increase in firefly luciferase expression when normalised to Renilla luciferase, the normalised expression ratios of firefly activity increases to 3.81 when unmethylated versus 0.0269 when methylated in vitro (Students *t*-test, *P* = 0.0057, *n* = 3) which corresponds to a 206-fold change increase in luciferase activity in the unmethylated *PSORS1C3* cloned vector compared to the methylated identical vector. *N* = 3 experimental repeats with each experiment containing three technical repeats. Error bars represent ± the standard error.

We found a marked increase in the relative expression of firefly luciferase activity normalised to *Renilla luciferase* in the unmethylated *PSORS1C3* cloned pCpGL vector compared to the methylated vector (Fig. 4) (fold change = 206, *P* = 0.006, *N* = 3). Next, we quantified gene expression levels of the *PSORS1C3* long non-coding gene in a cohort of 70 post-mortem brain samples (BA11, *N* = 38; BA25, *N* = 32, both regions obtained from the same individuals). Our analysis showed that in our sample set, *PSORS1C3* lncRNA was not expressed in the PFC (Ct Value >31 or undetermined). This result is consistent with findings in Genotype-Tissue Expression (GTEx) portal^48^ (https://www.gtexportal.org/home/gene/PSORS1C3), which shows little to no expression for this gene in brain samples examined. Next, we examined expression levels of the second closest gene (*POU5F1*) to the suicide-associated DMR. An LMM was used to compare mean dCt values between suicide cases and non-psychiatric controls and the analysis showed no significant difference in gene expression levels between the two groups (*P* = 0.598; Supplementary Fig. S8A**)**. Furthermore, we found no significant correlation between mean DNA methylation at the suicide-associated region *PSORS1C3* DMR and *POU5F1* gene expression levels (Pearson’s *R* = −0.04, *P* = 0.67; Supplementary Fig. S8B).

## Discussion

In this study, we utilised previously published and unpublished methylomic datasets to perform a meta-analysis of variable DNA methylation in the brain of suicide completers. DNA methylation data were available for two different brain regions (PFC: 4 cohorts, *N* = 211; CER: 3 cohorts, *N* = 114) and data were meta-analysed across the suicide cohorts for each brain region separately. To our knowledge, this represents the most extensive methylomic study of suicide completers using post-mortem brain tissue to date.

While several studies suggest the involvement of the PFC in SB^22,49–51^, suicide-associated epigenetic changes in the CER have not been investigated. However, the CER is known to play an important role in motor control, cognition, and emotional processing and is involved in a variety of psychiatric disorders, including depression, bipolar disorder, and schizophrenia^52^. Suicidal behaviour in those with depression has been associated with a decreased cerebellar volume^53^ and low regional cerebral blood flow in the cerebellum^54^. Moreover imaging studies have reported structural abnormalities associated to suicide attempt in MDD in cortical and subcortical regions, including cerebellum^55^ and alterations in functional cerebellum networks were found in depressed patients with a suicide attempt history^56^. Altogether these studies support the hypothesis of a potential involvement of CER in the psychopathology of attempted suicide in patients with MDD. We set out to further explore the role of DNA methylation and suicidality in this region as well as to determine if suicidality associated DNA methylation changes are brain region specific.

We first examined site-specific genome-wide patterns of DNA methylation in suicide cases compared with controls in the PFC and CER separately. We identified one DMP (*cg00963169, P* = 3.30E-08) in the PFC, which reached experiment-wide significance. This probe, located in the intronic region of the *ELAVL4* gene, shows consistent hypomethylation in suicide cases compared to controls. The *ELAVL4* gene has a known role in translation and stabilisation of mRNA, especially in the brain, and acts as a negative regulator of proliferation, activity, and differentiation in neural stem cells^57,58^. Through their mRNA stabilising activities, this family of proteins modulate neuronal development and maintenance, and their altered activity has been implicated in neurological conditions^59^ and disorders including Alzheimer’s disease^60^, schizophrenia^61^, and autism^62^. Of interest, the association with suicide at this site was found to be largely driven by PFC cohorts derived from neuronal nuclei (see Supplementary Fig. S5) and thus future replication of this finding in sorted neuronal cells may yield more significant associations with suicide.

In the CER, we identified six probes (cg14392966, cg17855963, cg25590492, cg12284382, cg10757978, cg04525580) at experiment-wide significance threshold (*P* < 1E-07). The top-ranked DMP, cg14392966 (*P* = 3.06E-11), is located in exon 1 of the *PUS3* gene. *PUS3* encodes a highly conserved enzyme responsible for post-transcriptional modification of tRNA and has previously been associated with intellectual disability^63^ and severe neurodevelopmental disorders^46^. The remaining DMPs include probes in the vicinity of several loci previously implicated in pathways relevant to psychiatric phenotypes. For example, *ZIC1* is thought to play an important role in neurogenesis and cerebellum differentiation^64^, whereas *RASD2* is known to modulate dopaminergic neurotransmission^65^. Furthermore, the probe cg04525580 (*P* = 9.08E-08) is located at the 5′UTR of the interferon regulatory factor 2 (*IRF2*) gene. This locus plays an important role in transcriptional activation at promoters^66^ and regulates the expression of a variety of genes involved in immune responses in the brain^67^, further supporting a role for immune-related pathways in suicide.

To increase the power of our study to identify changes in DNA methylation between cases and controls and given that DNA methylation at adjacent probes is often correlated, we employed the regional-based analysis, Comb-p, to identify DMRs. Our analysis identified three and eight significant (Sidak-corrected *P* < 0.05) DMRs in the PFC and CER, respectively. In the PFC, the top-ranked DMR, located in intron 1 of the *WRB* gene, is consistently hypomethylated across all five CpG sites in suicide cases relative to controls, in all cohorts. Recent studies suggest a role for Wrb in photoreceptor synaptic transmission in zebrafish^68^ and the *WRB* locus was reported among the differentially expressed genes in a mouse model study looking at cognitive impairment and neuropathology in Down syndrome brain^69^ further supporting the hypothesis of its involvement in the correct development and functioning of the CNS.

The second top-ranked suicide-associated DMR in the PFC (Sidak-corrected *P* = 3.81E-05) was located downstream the promoter region of the *PSORS1C3* non-coding gene, a DMR previously reported by our group as associated with MDD suicide completers^22^. Although the function of this gene product remains unclear, it is thought to be a potential regulator of nearby immune-related genes^70^ and is a known risk gene for psoriasis^71,72^, supporting a role in immune system regulation. To gain further insight into the role of this suicide-associated *PSORS1C3 DMR* on nearby gene expression, we first used a dual-luciferase assay to determine if DNA methylation at this region decreases expression of firefly luciferase in a CpG-free vector. The methylated *PSORS1C3 DMR* construct was significantly associated with decreased expression of the reporter gene product, indicating that the methylation status of this DMR has the potential to modify promoter activity; however, the identity of the modified gene product is unknown. We found no evidence of suicide-associated differential gene expression of nearby genes, *PSORS1C3* and *POU5F1*, in the PFC. We hypothesise that DNA methylation changes at this suicide-associated DMR are associated with a different nearby gene or an unknown splice variant of either the *PSORS1C3* or *POU5F1* genes. Finally, an additional DMR was identified in the PFC, located on chromosome 22, in the promoter region of the *LGALS1* gene. This gene is thought to play a role in immune system functioning^65^ and DNA methylation changes at this locus have previously been implicated in schizophrenia^33^.

In the CER, the top-ranked suicide-associated DMR, located in the intronic region of the *CERC2* gene on chromosome 22, spans four CpG sites. This locus is known to be involved in the control of the periodic oscillation of cyclin E expression in proliferating cells likely through its histone deacetylase activity^73^. The *CERC2*-associated DMR showed significant hypermethylation (Sidak-corrected *P* = 5.68E-07) across all four CpG sites within the region in suicide cases compared with controls and the direction of this change was found to be consistent across all three independent CER methylomic studies. To the best of our knowledge, this gene has not been previously implicated in the pathology of SB. Seven additional suicide-associated DMRs were identified in the CER. Of interest is the DMR located in exon 10 of the *SLC44A4* gene; a gene recently implicated in a study looking at the role of the major histocompatibility complex region in schizophrenia susceptibility^74^. An additional suicide-associated DMR, worthy of further investigation, is located in exon 3 of the *WWTR1* gene, a transcriptional coactivator known for its role in preserving neuronal health^75^. Furthermore, a missense variant in this gene was recently associated with lower cognitive ability in a GWAS study for infant mental and motor ability^76^. Finally, we identified a suicide-associated DMR located downstream the promoter region of *MED13L* gene and genetic variants at this locus have been widely reported as associated with intellectual disability^77,78^, suggesting that this gene may play an important role in neurological development.

Since SB is often a complication of a psychiatric disorder, distinguishing suicide diathesis-related DNA methylation changes from those associated with mood disorders and other psychiatric disorders has remained a challenge. In order to unravel the relative contribution of psychopathologies from DNA methylation changes specific to suicide, we performed an additional meta-analysis in the CER whereby individuals with documented Axis-I psychiatric disorders (MDD, SZ, BD) and who died by suicide were compared to psychiatric cases without a documented history of SB. Comparison of the results (effect size of the top 500 nominally significant (*P* < 0.05) DMPs) from our original CER meta-analysis and the secondary analysis revealed a strong positive correlation (*P* = 2.2E-16; *R* = 0.89). Moreover, we replicated our findings for two of the six CER-associated DMPs, which reached multiple testing threshold in our suicide cases versus psychiatric controls meta-analysis (cg10757978, *P* = 1.19E-04; cg04525580, *P* = 0.017) and we observed similar direction of effect for the remaining loci. Taken together these findings suggest that suicide-associated DMPs identified in the CER are largely independent of comorbid psychiatric disorders. Unfortunately, we were unable to perform the same analysis in the PFC due to the limited number of samples that did not die by suicide but had an Axis-I diagnosis.

Despite the power of the methodological approaches used in this study, there are several caveats. First, the modest number of studies included made this meta-analysis relatively underpowered to detect small changes in DNA methylation. Despite this we were able to identify several statistically significant DMPs and DMRs in both brain regions. Another major limitation is that bulk brain tissue was used in most of the studies included in our meta-analysis and cellular heterogeneity is a well-known confounder in DNA methylation studies. In order to bypass this issue, we used a previously reported in silico method to estimate the neuronal proportion in each sample in bulk PFC cohorts and included these estimates in the statistical models^32^. This method could not be applied to our analysis of the CER and thus it is plausible that cellular heterogeneity is confounding some of our CER results. Third, recent research has implicated the importance of other DNA modifications (i.e., 5-hydroxymethyl cytosine) in the brain^79^. Our measure of DNA methylation in this study cannot be distinguished from 5-hydroxymethyl cytosine (5hmC). Of interest, we examined the presence of detectable 5hmC levels at statistical significant DMPs identified in this study in their respective brain regions using the Hydroxymethylation Annotation in Brain Integrative Tool (HABIT) tool (http://epigenetics.iop.kcl.ac.uk/HMC/)^41^. This tool identified detectable levels of 5hmC at the following suicide-associated DMPs cg00963169 (PFC), and cg17855963 and cg04525580 (CER), suggesting that the majority of DMPs identified in this study are not confounded by 5hmC. However, future studies should attempt to examine the role of 5hmC in SB. Fourth, medication data, smoking information, and method of suicide were not available for all individuals; thus, we cannot rule out the possibility that the observed DNA methylation changes are influenced by these potential confounders.

Fifth, we acknowledge the possibility that many of the associations reported (DMPs/DMRs) could be related to the severity and/or duration of the mental health disorder. From our secondary analysis in the cerebellum we show that for certain top-ranked DMPs the association appears to be suicide associated rather than associated with the underlying mental health disorder. Given the lack of information related to severity and/or duration of mental illness for samples included in this meta-analysis we cannot rule out the contribution of the above-mentioned confounders.

Finally, although our study presents evidence for novel DNA methylation changes associated with suicide, further replication using a larger sample size is required to support these results. In addition, future studies could also examine the transcriptional consequences of the observed DNA methylation changes at the *PSORS1C3 DMR* on additional nearby genes and/or novel splice variants in the region. There is considerable interest in using DNA methylation-based biomarkers as predictors for suicide risk and previous studies^30,80–84^ have identified polymorphic CpGs that can act as a unique molecular signature for suicide prediction. The data from this study provide many more candidate regions as potential biomarkers for suicide risk and also identifies genes/networks potentially dysregulated in suicidal brain.

In summary, our data, which utilise several published and unpublished suicide cohorts, have identified DMPs and several DMRs associated with suicide in both the PFC and CER, including the previously identified DMR upstream of the *PSORS1C3* non-coding gene. We show that this DMR can influence gene expression using a dual-luciferase assay, but we have yet to identify its target gene.

## Supplementary information

Supplementary Figure S1

Supplementary Figure S2

Supplementary Figure S3

Supplementary Figure S4

Supplementary Figure S5

Supplementary Figure S6

Supplementary Figure S7

Supplementary Figure S8

Suppelementary Table S1

Suppelementary Table S2

Suppelementary Table S3

Suppelementary Table S4

Suppelementary Table S5

Suppelementary Table S6

Suppelementary Table S7
