# Supplementary figures and images for: Genome-wide DNA methylation meta-analysis in the brains of suicide completers
Source: Transl Psychiatry. 2020 Feb 19;10:69. doi: 10.1038/s41398-020-0752-7 (PMC7031296; doi:10.1038/s41398-020-0752-7)

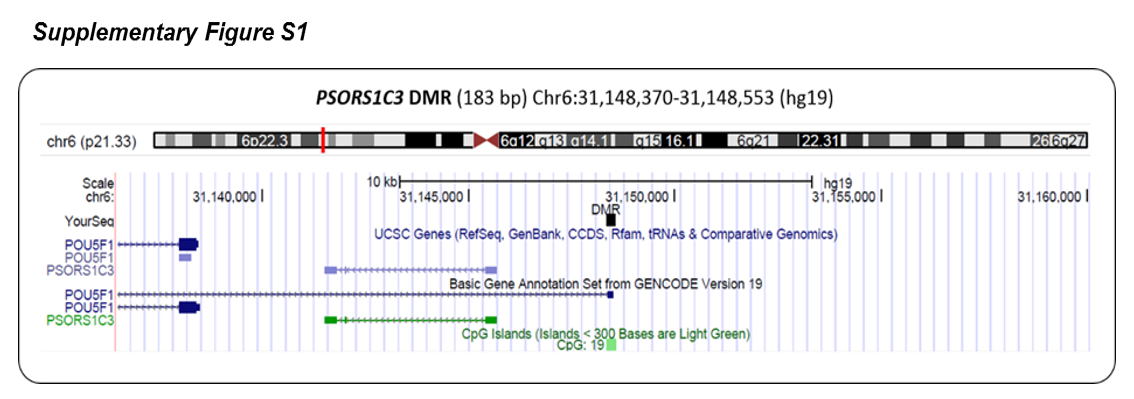

Supplement: Supplementary file 1 — Supplementary Figure S1 [file 41398_2020_752_MOESM1_ESM.tif]

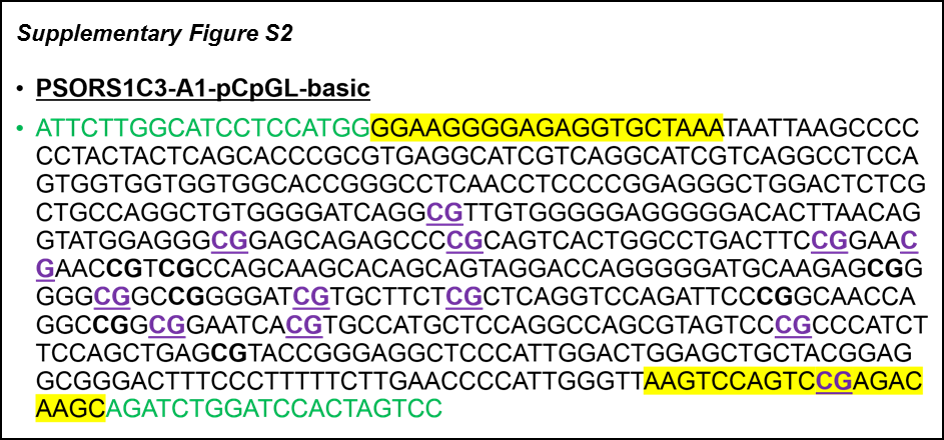

Supplement: Supplementary file 2 — Supplementary Figure S2 [file 41398_2020_752_MOESM2_ESM.tif]

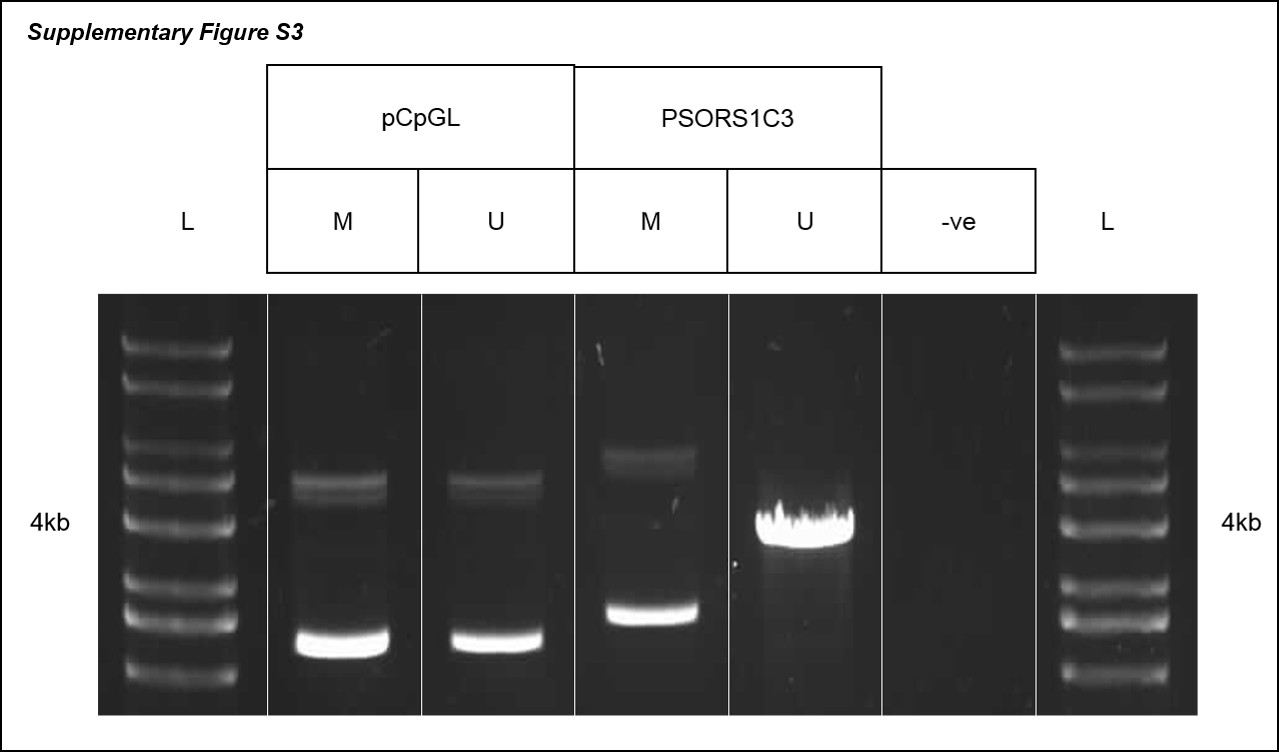

Supplement: Supplementary file 3 — Supplementary Figure S3 [file 41398_2020_752_MOESM3_ESM.tif]

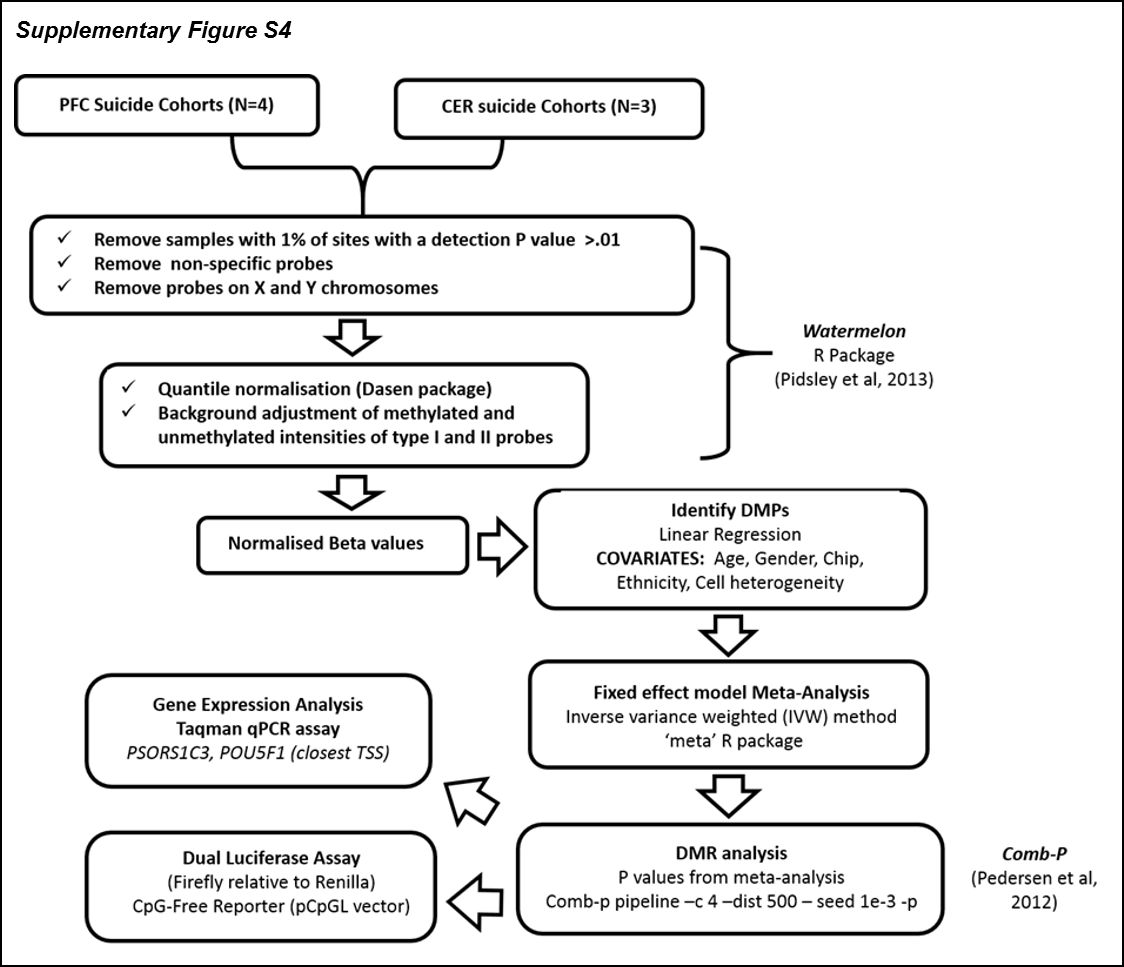

Supplement: Supplementary file 4 — Supplementary Figure S4 [file 41398_2020_752_MOESM4_ESM.tif]

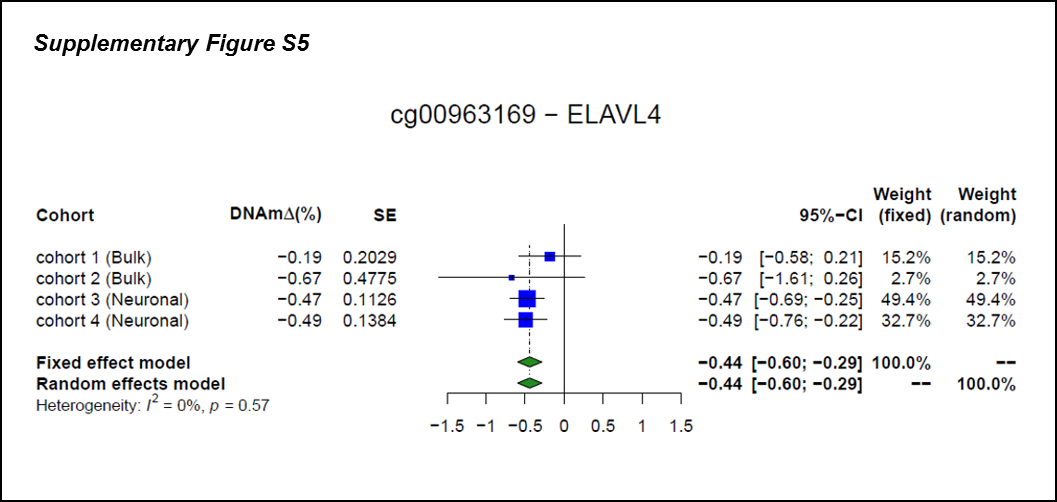

Supplement: Supplementary file 5 — Supplementary Figure S5 [file 41398_2020_752_MOESM5_ESM.tif]

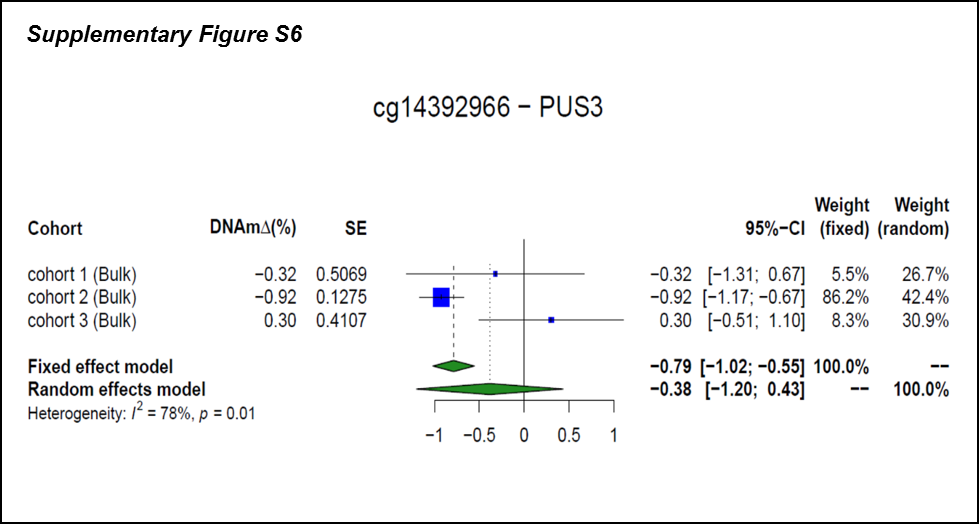

Supplement: Supplementary file 6 — Supplementary Figure S6 [file 41398_2020_752_MOESM6_ESM.tif]

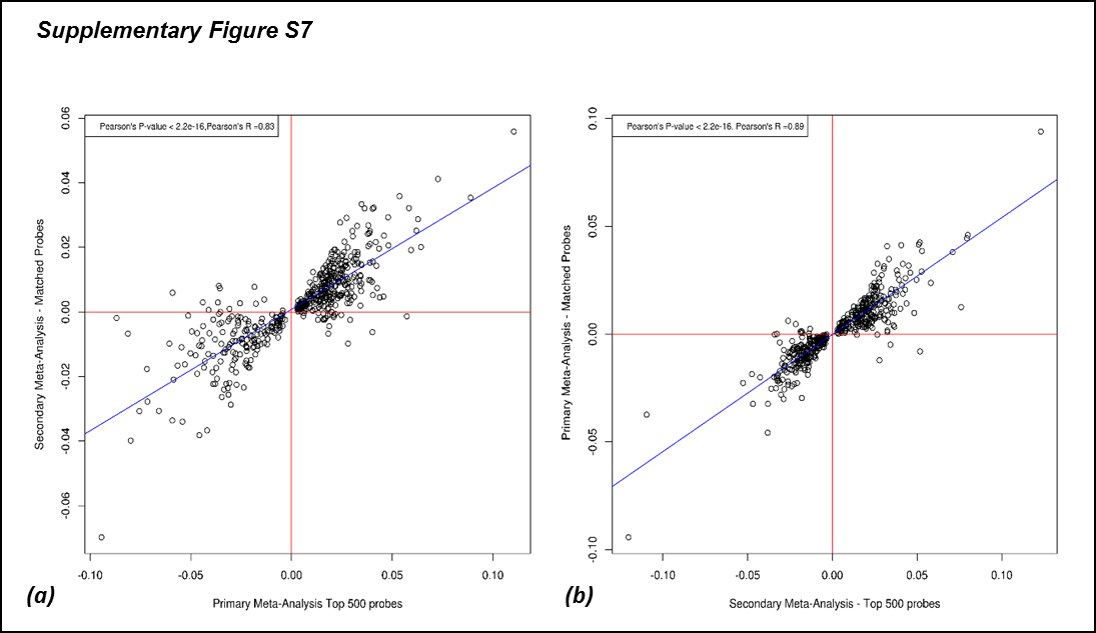

Supplement: Supplementary file 7 — Supplementary Figure S7 [file 41398_2020_752_MOESM7_ESM.tif]

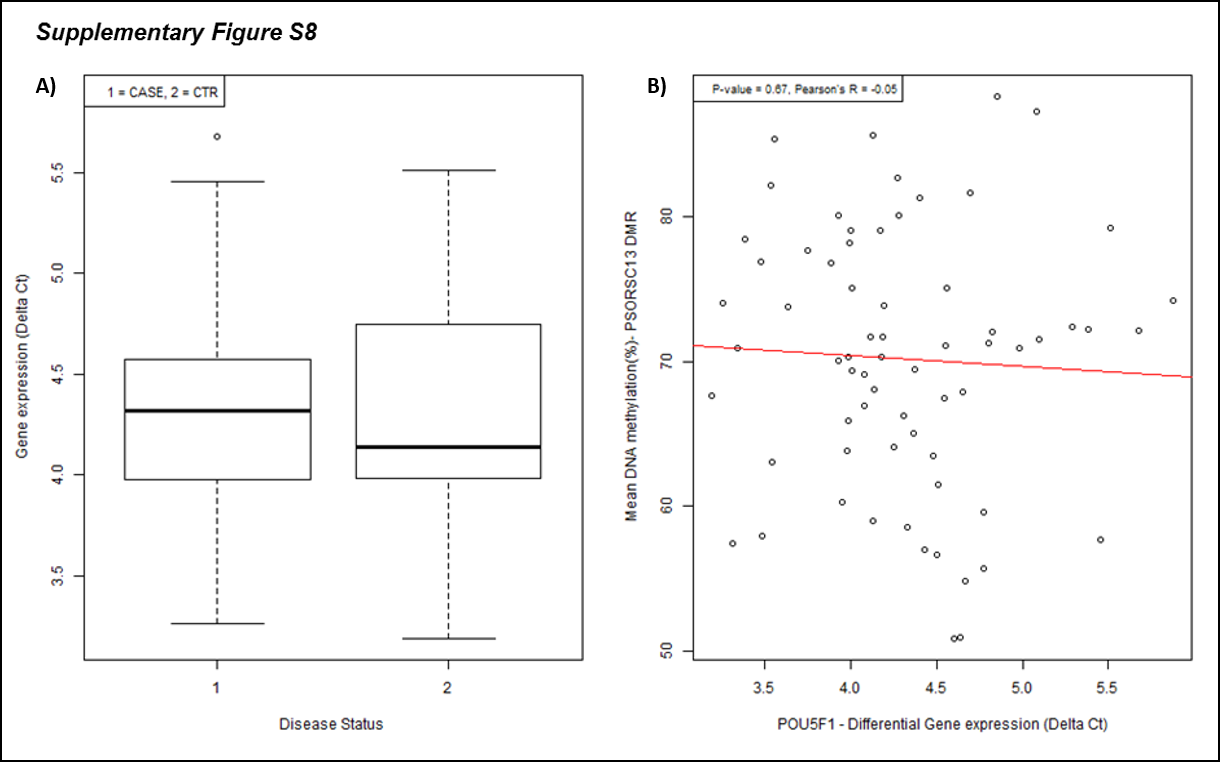

Supplement: Supplementary file 8 — Supplementary Figure S8 [file 41398_2020_752_MOESM8_ESM.tif]
