# Supplementary material for: Genome-wide DNA methylation meta-analysis in the brains of suicide completers
Source: Transl Psychiatry. 2020 Feb 19;10:69. doi: 10.1038/s41398-020-0752-7 (PMC7031296; doi:10.1038/s41398-020-0752-7)
Supplement: Supplementary file 9 — Suppelementary Table S1 [file 41398_2020_752_MOESM9_ESM.docx]

| *Supplementary Table S1*. Demographics summary of discovery cohorts | | | | | | | | | |
| --- | --- | --- | --- | --- | --- | --- | --- | --- | --- |
| *Prefrontal Cortex* | **Cohort** | **N** | **Age at death (Yrs)** | **Gender** | | **PMI (hrs)** | **Brain weight (g)** | **pH** | **Neuronal Comp.** |
|  |  |  | Mean (SD) | M | F | Mean (SD) | Mean (SD) | Mean (SD) | Mean (SD) |
|  | ***Viana J et al, 2017*** |  |  |  |  |  |  |  |  |
|  | Suicide Cases | 11 | 31.5 (9.3) | 10 | 1 | 56.2 (33) | NA | 13.1 (6.6) | 0.3 (0.1) |
|  | Non-Psychiatric Controls | 24 | 43.6 (13.4) | 20 | 4 | 41.6 (20) | NA | 8.9 (6.8) | 0.3 (0.1) |
|  | **Total** | 35 |  |  |  |  |  |  |  |
|  | ***Guintivano J et al, 2013*** |  |  |  |  |  |  |  |  |
|  | Suicide Cases | 22 | 27.4 (16.6) | 12 | 10 | 19.3 (6.5) | NA | NA | Neuronal |
|  | Non-Psychiatric Controls | 35 | 35.2 (14.9) | 16 | 19 | 15.9 (5.7) | NA | NA | Neuronal |
|  | **Total** | 57 |  |  |  |  |  |  |  |
|  | ***Kozlenkov et al, 2017*** |  |  |  |  |  |  |  |  |
|  | Suicide Cases | 21 | 31.5 (10.1) | 16 | 6 | 4.1 (0.2) | NA | 6.9 (0.1) | Neuronal |
|  | Non-Psychiatric Controls | 29 | 37.4 (13.7) | 18 | 5 | 4 (1) | NA | 6.7 (0.2) | Neuronal |
|  | **Total** | 50 |  |  |  |  |  |  |  |
|  | ***Murphy et al, 2017*** |  |  |  |  |  |  |  |  |
|  | ***BA11*** |  |  |  |  |  |  |  |  |
|  | Suicide Cases | 20 | 48.6 (20.8) | 5 | 15 | 20 (15.6) | NA | 6.6 (0.3) | 0.45 (0.06) |
|  | Non-Psychiatric Controls | 20 | 39.4 (19.5) | 4 | 16 | 26.1 (20.5) | NA | 5.8 (3.5) | 0.43(0.11) |
|  | **Total** | 40 |  |  |  |  |  |  |  |
|  | ***BA25*** |  |  |  |  |  |  |  |  |
|  | Suicide Cases | 17 | 49.5 (22.4) | 4 | 13 | 20.2 (16.9) | NA | 6.5 (0.2) | 0.33 (0.09) |
|  | Non-Psychiatric Controls | 18 | 41.2 (19.6) | 4 | 14 | 23.4 (18.7) | NA | 5.7(3.7) | 0.33 (0.13) |
|  | **Total** | 35 |  |  |  |  |  |  |  |
| *Cerebellum* | ***Viana e al, 2017*** |  |  |  |  |  |  |  |  |
|  | Suicide Cases | 7 | 32.6 (9.4) | 6 | 1 | 55.9 (34.5) | NA | 6.6 (0.4) | NA |
|  | Non-Psychiatric Controls | 16 | 44.1 (16.2) | 13 | 3 | 32.2 (16.1) | NA | 6.5 (0.3) | NA |
|  | **Total** | 23 |  |  |  |  |  |  |  |
|  | ***Stanley Brain Bank 1*** |  |  |  |  |  |  |  |  |
|  | Suicide Cases | 18 | 38.8 (10.9) | 12 | 7 | 29.6 (16.2) | NA | 6.2 (0.2) | NA |
|  | Non-Psychiatric Controls | 15 | 48.1 (10.6) | 9 | 6 | 23.7 (9.9) | NA | 6.3 (0.2) | NA |
|  | **Total** | 33 |  |  |  |  |  |  |  |
|  | ***Stanley Brain Bank 2*** |  |  |  |  |  |  |  |  |
|  | Suicide Cases | 21 | 42.8 (10.1) | 10 | 11 | 37.6 (18.2) | 1450.8 (121.5) | 6.9 (0.1) | NA |
|  | Non-Psychiatric Controls | 37 | 45.2 (9.3) | 27 | 11 | 30.7 (15.1) | 1435.2 (150.7) | 6.6 (0.3) | NA |
|  | Total | 58 |  |  |  |  |  |  |  |
| *Abbreviations*: Prefrontal Cortex, PFC; Cerebellum, CER; Post-mortem Interval, PMI; Standard deviation, SD; Broadmann Area 11, BA11; Broadmann Area 25, BA25. | | | | | | | | | |
