# Supplementary material for: Genome-wide DNA methylation meta-analysis in the brains of suicide completers
Source: Transl Psychiatry. 2020 Feb 19;10:69. doi: 10.1038/s41398-020-0752-7 (PMC7031296; doi:10.1038/s41398-020-0752-7)
Supplement: Supplementary file 10 — Suppelementary Table S2 [file 41398_2020_752_MOESM10_ESM.docx]

***Supplementary Table S2***. Pre-optimized TaqMan ® Assays for targeted gene expression analysis.

| **Target Gene Symbol** | **Target Gene Name** | **Catalog number** | **Assay ID** | **Chromosome Location (GRCh38)** | **Assay Design** | **Exon boundary** | **Assay location** | **Amplicon Length** |
| --- | --- | --- | --- | --- | --- | --- | --- | --- |
| PSORS1C3 | Psoriasis Susceptibility 1 Candidate 3 | 4351372 | Hs05014866_m1 | Chr.6: 31173735 - 31177899 | Probe spans exons | 1-2 | 294 | 83 |
| POU5F1 | POU Class 5 Homeobox 1 | 4331182 | Hs04260367_gH | Chr.6: 31164337 - 31170693 | Both primers and probe map within a single exon | 5-5 | 1400 | 77 |
| CYC1 | Cytochrome C1 | 4331182 | Hs00357717_m1 | Chr.8: 144095035 - 144097527 | Probe spans exons | 5-6 | 832 | 73 |
| RPL13 | Ribosomal Protein L13 | 4331182 | Hs00744303_s1 | Chr.16: 89560657 - 89566829 | Both primers and probe map within a single exon | 6-6 | 866 | 137 |
| UBE2D2 | Ubiquitin Conjugating Enzyme E2 D2 | 4331182 | Hs00366152_m1 | Chr.5: 139561166 - 139628434 | Probe spans exons | 5-6 | 937 | 70 |
| *Abbreviations*: GRCh38, Genome Reference Consortium Human genome build 38. | | | | | | | | |
