# Supplementary material for: Genome-wide DNA methylation meta-analysis in the brains of suicide completers
Source: Transl Psychiatry. 2020 Feb 19;10:69. doi: 10.1038/s41398-020-0752-7 (PMC7031296; doi:10.1038/s41398-020-0752-7)
Supplement: Supplementary file 11 — Suppelementary Table S3 [file 41398_2020_752_MOESM11_ESM.docx]

| ***Supplementary Table S3.* Top 20 suicide-associated DMPs in human PFC** | | | | | | | | |
| --- | --- | --- | --- | --- | --- | --- | --- | --- |
| **Probe ID** | **Mean  Δβ** | **P-value (Fixed Effect)** | **CHR** | **Hg19** | **Illumina Annotation (UCSC Ref Gene Name)** | **Probe**  **Type** | **Gene annotation from GREAT (Distance from TSS)** | **SNP in Probe Sequence (>10bp from SBE)** |
| cg00963169 | -0.44 | 3.30E-08 | 1 | Chr1:50513927 | *ELAVL4* | I | *ELAVL4* (-60666), *AGBL4* (-24302) |  |
| cg20963696 | 1.06 | 3.78E-06 | 5 | Chr5:10679606 | *DAP* | II | *DAP* (+81780), *ROPN1L* (+237633) |  |
| cg15362194 | -0.30 | 3.92E-06 | 2 | Chr2:98262546 | *COX5B* | I | *COX5B* (+26) |  |
| cg18663341 | -0.86 | 5.06E-06 | 7 | Chr7:45148416 | *TBRG4* | II | *NACAD* (-19924), *TBRG4* (+2900) |  |
| cg19477433 | -1.08 | 5.15E-06 | 6 | Chr6:168530427 |  | II | *FRMD1* (-50589), *DACT2* (+189974) |  |
| cg20705321 | -1.07 | 5.52E-06 | 19 | Chr19:10958970 | *C19orf38* | I | *C19orf38* (-135) |  |
| cg07951810 | 0.57 | 5.79E-06 | 12 | Chr12:65153561 | *GNS* | II | *GNS* (-336) |  |
| cg15570432 | -0.86 | 7.33E-06 | 20 | Chr20:30460881 | *TTLL9* | II | *DUSP15* (-2403), *TTLL9* (+2377) |  |
| cg08847533 | -0.66 | 9.46E-06 | 14 | Chr14:75593920 | *NEK9* | I | *NEK9* (-143) | rs12889309 |
| cg00264958 | -0.84 | 1.44E-05 | 17 | Chr17:48023189 |  | II | *TAC4* (-97811), *DLX4* (-23372) | rs12937307 |
| cg00409434 | -1.54 | 1.59E-05 | 2 | Chr2:46414317 | *PRKCE* | II | *EPAS1* (-110223), *PRKCE* (+535275) |  |
| cg26011314 | -0.86 | 1.86E-05 | 6 | Chr6:129255129 | *LAMA2* | II | *LAMA2* (+50844), *ARHGAP18* (+776240) |  |
| ch.1.3947147F | -2.62 | 1.86E-05 | 1 | Chr1:203693763 | *ATP2B4* | II | *LAX1* (-40520), *ATP2B4* (+97849) |  |
| cg18392139 | -1.20 | 1.89E-05 | 5 | Chr5:56719150 |  | II | *ACTBL2* (+59485), *GPBP1* (+209250) |  |
| cg18100887 | 6.38 | 1.98E-05 | 17 | Chr17:34611341 |  | II | *TBC1D3C* (-19310), *CCL3L1* (+14388) |  |
| cg03976172 | -1.76 | 1.99E-05 | 8 | Chr8:106666746 | *ZFPM2* | II | *OXR1* (-793405), *ZFPM2* (+335600) |  |
| cg02522906 | -1.12 | 2.02E-05 | 10 | Chr10:123450196 |  | II | *FGFR2* (-92225), *ATE1* (+237349) | rs2132902 |
| cg19326874 | -1.45 | 2.06E-05 | 4 | Chr4:37903243 | *TBC1D1* | II | *PTTG2* (-58812), *TBC1D1* (+10539) |  |
| cg21712019 | -0.39 | 2.21E-05 | 19 | Chr19:50316342 | *FUZ* | I | *FUZ* (+224) |  |
| cg17872838 | 1.65 | 2.22E-05 | 13 | Chr13:52607339 | *UTP14C* | II | *UTP14C* (+4399), *NEK5* (+95874) |  |
| Abbreviations: PFC, Pre-frontal cortex; DMPs, differentially methylated positions; Hg19, Human Genome version 19; GREAT, Genomic Regions Enrichment of Annotations Tool; TSS, transcription start site; UCSC, University of California, Santa Cruz Genome Browser, SBE, Single binding extension. | | | | | | | | |
