# Supplementary material for: Genome-wide DNA methylation meta-analysis in the brains of suicide completers
Source: Transl Psychiatry. 2020 Feb 19;10:69. doi: 10.1038/s41398-020-0752-7 (PMC7031296; doi:10.1038/s41398-020-0752-7)
Supplement: Supplementary file 12 — Suppelementary Table S4 [file 41398_2020_752_MOESM12_ESM.docx]

| ***Supplementary Table S4.* Top 20 suicide-associated DMPs in human CER** | | | | | | | | |
| --- | --- | --- | --- | --- | --- | --- | --- | --- |
| **Probe ID** | **Mean Δβ** | ***P-*value (Fixed Effect)** | **CHR** | **Hg19** | **Illumina annotation** | **Probe  Type** | **Gene annotation from GREAT (Distance from TSS)** | **SNP in Probe Sequence  (>10bp from SBE)** |
| cg14392966 | -0.786 | 3.06E-11 | 11 | Chr11:125773125 | *DDX25;PUS3* | I | *DDX25* (-1146), *PUS3* (-10) |  |
| cg17855963 | 1.644 | 5.37E-10 | 6 | Chr6:15873800 |  | II | *MYLIP* (-255516), *DTNBP1* (-210512) |  |
| cg25590492 | -1.504 | 2.22E-09 | 3 | Chr3:147129952 | *ZIC1* | II | *ZIC1* (+2772) |  |
| cg12284382 | -2.662 | 5.92E-09 | 22 | Chr22:35940438 | *RASD2* | II | *RASD2* (+3087), *MB* (+72945) |  |
| cg10757978 | 3.261 | 5.84E-08 | 8 | Chr8:70623125 | *SLCO5A1* | II | *SULF1* (+83119), *SLCO5A1* (+124173) | rs2933044 |
| cg04525580 | 1.156 | 9.08E-08 | 4 | Chr4:185352181 | *IRF2* | II | *ENPP6* (-213068), *IRF2* (+43544) |  |
| cg08087379 | 4.569 | 1.08E-07 | 22 | Chr22:31364274 | *TUG1;MORC2* | II | *MORC2* (-88) |  |
| cg08982904 | 2.912 | 1.34E-07 | 8 | Chr8:43127238 |  | I | *HGSNAT* (+131647) | rs62516805 |
| cg24197303 | -1.704 | 1.35E-07 | 7 | Chr7:157572599 | *PTPRN2* | II | *DNAJB6* (+442890), *PTPRN2* (+807882) |  |
| cg07419968 | -1.686 | 1.49E-07 | 1 | Chr1:106339081 |  | II | NONE |  |
| cg13702370 | 6.426 | 1.64E-07 | 11 | Chr11:45844932 |  | II | *CRY2* (-24024), *SLC35C1* (+19310) |  |
| cg13606991 | 6.278 | 1.87E-07 | 1 | Chr1:182556113 | *RNASEL* | II | *RNASEL* (+2280), *RGSL1* (+136858) |  |
| cg17330203 | 1.708 | 2.09E-07 | 10 | Chr10:101515599 | *CUTC* | II | *ABCC2* (-26863), *COX15* (-23177) |  |
| cg22230538 | 0.595 | 2.49E-07 | 8 | Chr8:28258841 |  | I | *FZD3* (-92880), *ZNF395* (-14865) |  |
| cg24690692 | -5.011 | 3.55E-07 | 7 | Chr7:123842309 |  | II | *TMEM229A* (-168787), *GPR37* (+563371) |  |
| cg01767927 | 1.855 | 4.47E-07 | 17 | Chr17:78821024 | *RPTOR* | II | *CHMP6* (-144616), *RPTOR* (+302400) |  |
| cg06791979 | -7.151 | 5.25E-07 | 17 | Chr17:75474070 | *SEPT9* | II | *TNRC6C* (-526247), *SEPT9* (+196579) |  |
| cg03914925 | 1.712 | 6.75E-07 | 6 | Chr6:170405976 |  | II | *DLL1* (+193720), *C6orf70* (+254256) |  |
| cg10548399 | 2.184 | 1.04E-06 | 8 | Chr8:143009151 |  | II | *FLJ43860* (-491822), *TSNARE1* (+475391) |  |
| cg02464608 | 2.144 | 1.06E-06 | 3 | Chr3:122631723 | *SEMA5B* | II | *SEMA5B* (+114852), *DIRC2* (+117823) |  |
| *Abbreviations:* CER, cerebellum; DMPs, differentially methylated positions; Hg19, Human Genome version 19; GREAT, Genomic Regions Enrichment of Annotations Tool; TSS, transcription start site; UCSC, University of California Santa Cruz Genome Browser; SBE, Single binding extension. | | | | | | | | |
