# Supplementary material for: Genome-wide DNA methylation meta-analysis in the brains of suicide completers
Source: Transl Psychiatry. 2020 Feb 19;10:69. doi: 10.1038/s41398-020-0752-7 (PMC7031296; doi:10.1038/s41398-020-0752-7)
Supplement: Supplementary file 13 — Suppelementary Table S5 [file 41398_2020_752_MOESM13_ESM.docx]

| ***Supplementary Table S5:* Gene Ontology PFC meta-analysis Suicide-associated DMPs (P-value= 1E-4)** | | | | | | | | | | | |
| --- | --- | --- | --- | --- | --- | --- | --- | --- | --- | --- | --- |
| **Independent pathways** | | | | | | | | | **Non-independent pathways** | | |
| **ID** | **Name** | **Type** | **nProbesinPathway** | **nGenesinPathway** | **nTestListProbesinPathway** | **nTestListGenesinPathway** | **P.GenesinTestList** | **GenesinTestListAndPathway** | **ID** | **Name** | **Type** |
| GO:0009206 | purine ribonucleoside triphosphate biosynthetic process | biological_process | 1845 | 50 | 3 | 3 | 2.53E-09 | COX5B\|PRKAG2\|TXNDC3 | GO:0009145 | purine nucleoside triphosphate biosynthetic process | Biological Process |
|  |  |  |  |  |  |  |  |  | GO:0009201 | ribonucleoside triphosphate biosynthetic process | Biological Process |
|  |  |  |  |  |  |  |  |  | GO:0009142 | nucleoside triphosphate biosynthetic process | Biological Process |
|  |  |  |  |  |  |  |  |  | GO:0009152 | purine ribonucleotide biosynthetic process | Biological Process |
|  |  |  |  |  |  |  |  |  | GO:0006164 | purine nucleotide biosynthetic process\| | Biological Process |
|  |  |  |  |  |  |  |  |  | GO:0009260 | ribonucleotide biosynthetic process | Biological Process |
| GO:0030317 | sperm motility | biological_process | 1570 | 69 | 3 | 3 | 1.92E-08 | ATP2B4\|ING2\|TXNDC3 | GO:0097722 | sperm motility | Biological Process |
| GO:0097228 | sperm principal piece | cellular_component | 937 | 21 | 2 | 2 | 3.96E-08 | ATP2B4\|TXNDC3 |  |  |  |
| GO:0035640 | exploration behavior | biological_process | 806 | 24 | 2 | 2 | 9.30E-08 | ABAT\|PRKCE |  |  |  |
| GO:0021549 | cerebellum development | biological_process | 4045 | 87 | 3 | 3 | 3.49E-07 | ABAT\|HSPA5\|RFX4 | GO:0022037 | metencephalon development | Biological Process |
| GO:0021587 | cerebellum morphogenesis | biological_process | 1601 | 34 | 2 | 2 | 9.09E-07 | HSPA5\|RFX4 | GO:0021575 | hindbrain morphogenesis | Biological Process |
|  |  |  |  |  |  |  |  |  | GO:0021695 | cerebellar cortex development | Biological Process |
| GO:0006754 | ATP biosynthetic process | biological_process | 1496 | 38 | 2 | 2 | 1.81E-06 | COX5B\|PRKAG2 |  |  |  |
| GO:0043279 | response to alkaloid | biological_process | 5140 | 110 | 3 | 3 | 2.32E-06 | ABAT\|HSPA5\|PRKCE |  |  |  |
| GO:0050806 | positive regulation of synaptic transmission | biological_process | 7420 | 124 | 3 | 3 | 5.91E-06 | ABAT\|LAMA2\|PRKCE |  |  |  |
| GO:0060359 | response to ammonium ion | biological_process | 6063 | 125 | 3 | 3 | 6.22E-06 | ABAT\|HSPA5\|PRKCE |  |  |  |
