# Supplementary material for: Genome-wide DNA methylation meta-analysis in the brains of suicide completers
Source: Transl Psychiatry. 2020 Feb 19;10:69. doi: 10.1038/s41398-020-0752-7 (PMC7031296; doi:10.1038/s41398-020-0752-7)
Supplement: Supplementary file 14 — Suppelementary Table S6 [file 41398_2020_752_MOESM14_ESM.docx]

| ***Supplementary Table S6*: Gene Ontology CER meta-analysis Suicide-associated DMPs (*P*-value= 1E-4)** | | | | | | | | | | | |
| --- | --- | --- | --- | --- | --- | --- | --- | --- | --- | --- | --- |
| **Independent pathways** | | | | | | | | | **Non independent pathways** | | |
| **ID** | **Name** | **Type** | **nProbesinPathway** | **nGenesinPathway** | **nTestListProbesinPathway** | **nTestListGenesinPathway** | **P.GenesinTestList** | **GenesinTestListAndPathway** | **ID** | **Name** | **Type** |
| GO:1900452 | regulation of long term synaptic depression | biological_process | 415 | 12 | 3 | 3 | 1.46E-07 | DGKI\|SHANK3\|SORCS3 |  |  |  |
| GO:0043548 | phosphatidylinositol 3-kinase binding | molecular_function | 1214 | 29 | 4 | 4 | 4.08E-07 | CBL\|FYN\|PIK3AP1\|RASD2 |  |  |  |
| GO:0046621 | negative regulation of organ growth | biological_process | 1554 | 28 | 4 | 4 | 5.54E-07 | KCNK2\|PTK2\|TBX5\|TP73 | GO:0055022 | negative regulation of cardiac muscle tissue growth |  |
|  |  |  |  |  |  |  |  |  | GO:0061117 | negative regulation of heart growth |  |
| GO:0060044 | negative regulation of cardiac muscle cell proliferation | biological_process | 966 | 13 | 3 | 3 | 6.05E-07 | KCNK2\|TBX5\|TP73 |  |  |  |
| GO:0006555 | methionine metabolic process | biological_process | 654 | 20 | 3 | 3 | 3.90E-06 | AHCYL2\|CTH\|MAT1A |  |  |  |
| GO:0034236 | protein kinase A catalytic subunit binding | molecular_function | 1967 | 14 | 3 | 3 | 5.22E-06 | KCNQ1\|PRKAR1B\|SOX9 |  |  |  |
