# Supplementary material for: Genome-wide DNA methylation meta-analysis in the brains of suicide completers
Source: Transl Psychiatry. 2020 Feb 19;10:69. doi: 10.1038/s41398-020-0752-7 (PMC7031296; doi:10.1038/s41398-020-0752-7)
Supplement: Supplementary file 15 — Suppelementary Table S7 [file 41398_2020_752_MOESM15_ESM.docx]

| ***Supplementary Table S7***. **Secondary Meta-Analysis in CER cohorts – Suicide-specific DMPs** | | | | | | | | | |
| --- | --- | --- | --- | --- | --- | --- | --- | --- | --- |
| **Probe ID** | **Illumina Annotation (UCSC RefGene Name)** | **CHR** | **Hg19** | **Probe**  **Type** | **Gene annotation from GREAT (Distance from TSS)** | **Suicide *vs* Control (Mean Δβ)** | **Suicide *vs* Control (*P* value)** | **Suicide *vs* Psychiatric  (Mean Δβ)** | **Suicide *vs* Psychiatric  (*P* value)** |
| cg14392966 | *DDX25;PUS3* | 11 | Chr11:125773125 | I | *DDX25* (-1146), *PUS3* (-10) | -0.79 | 3.06E-11 | -0.16 | 3.24E-01 |
| cg17855963 |  | 6 | Chr6:15873800 | II | *MYLIP* (-255516), *DTNBP1* (-210512) | 1.64 | 5.37E-10 | 0.75 | 8.61E-02 |
| cg25590492 | *ZIC1* | 3 | Chr3:147129952 | II | *ZIC1* (+2772) | -1.50 | 2.22E-09 | -0.32 | 1.89E-01 |
| cg12284382 | *RASD2* | 22 | Chr22:35940438 | II | *RASD2* (+3087), *MB* (+72945) | -2.66 | 5.92E-09 | -0.27 | 5.44E-01 |
| cg10757978 | *SLCO5A1* | 8 | Chr8:70623125 | II | *SULF1* (+83119), *SLCO5A1* (+124173) | 3.26 | 5.84E-08 | 2.66 | 1.19E-04 |
| cg04525580 | *IRF2* | 4 | Chr4:185352181 | II | *ENPP6* (-213068), *IRF2* (+43544) | 1.16 | 9.08E-08 | 0.76 | 1.69E-02 |
| **Abbreviations**: CER, cerebellum;Hg19, Human Genome version 19; GREAT, Genomic Regions Enrichment of Annotations Tool; TSS, transcription start site; UCSC,  University of California Santa Cruz Genome Browser; DMPs, differentially methylated probes. | | | | | | | | | |
